# Pathological and clinical features of multiple cancers and lung adenocarcinoma: a multicentre study

**DOI:** 10.1093/icvts/ivac047

**Published:** 2022-02-21

**Authors:** Pietro Bertoglio, Luigi Ventura, Vittorio Aprile, Maria Angela Cattoni, Dania Nachira, Filippo Lococo, Maria Rodriguez Perez, Francesco Guerrera, Fabrizio Minervini, Letizia Gnetti, Alessandra Lenzini, Francesca Franzi, Giulia Querzoli, Guido Rindi, Salvatore Bellafiore, Federico Femia, Giuseppe Salvatore Bogina, Diana Bacchin, Peter Kestenholz, Enrico Ruffini, Massimiliano Paci, Stefano Margaritora, Andrea Selenito Imperatori, Marco Lucchi, Luca Ampollini, Alberto Claudio Terzi

**Affiliations:** 1 Division of Thoracic Surgery, IRCCS Azienda Ospedaliero-Universitaria of Bologna, Bologna, Italy; 2 Division of Thoracic Surgery, University Hospital of Parma, Parma, Italy; 3 Division of Thoracic Surgery, University Hospital of Pisa, Pisa, Italy; 4 Division of Thoracic Surgery, University of Insubria, Varese, Italy; 5 Department of General Thoracic Surgery, Fondazione Policlinico “A. Gemelli”-Catholic University of Sacred Heart, Rome, Italy; 6 Division of Thoracic Surgery, Clinica Universidad de Navarra, Madrid, Spain; 7 Division of Thoracic Surgery, University of Torino, Torino, Italy; 8 Division of Thoracic Surgery. Cantonal Hospital Lucerne, Lucerne, Switzerland; 9 Division of Pathological Anatomy, University Hospital of Parma, Parma, Italy; 10 Division of Pathological Anatomy, University of Insubria, Varese, Italy; 11 Division of Pathological Anatomy, IRCCS Sacro Cuore Don Calabria Hospital, Verona, Italy; 12 Division of Pathological Anatomy, Fondazione Policlinico “A. Gemelli”-Catholic University of Sacred Heart, Rome, Italy; 13 Division of Pathological Anatomy, Azienda USL di Reggio Emilia-IRCCS, Reggio Emilia, Italy; 14 Division of Thoracic Surgery, IRCCS Sacro Cuore Don Calabria Hospital, Verona, Italy; 15 Division of Thoracic Surgery, Azienda USL di Reggio Emilia-IRCCS, Reggio Emilia, Italy

**Keywords:** Lung adenocarcinoma, Multiple cancers, Adenocarcinoma subtype, Lung cancer, Thoracic surgery

## Abstract

**OBJECTIVES:**

Lung cancer is increasingly diagnosed as a second cancer. Our goal was to analyse the characteristics and outcomes of early-stage resected lung adenocarcinomas in patients with previous cancers (PC) and correlations with adenocarcinoma subtypes.

**METHODS:**

We retrospectively reviewed data of patients radically operated on for stage I–II lung adenocarcinoma in 9 thoracic surgery departments between 2014 and 2017. Overall survival (OS) and time to disease relapse were evaluated between subgroups.

**RESULTS:**

We included 700 consecutive patients. PC were present in 260 (37.1%). Breast adenocarcinoma, lung cancer and prostate cancer were the most frequent (21.5%, 11.5% and 11.2%, respectively). No significant differences in OS were observed between the PC and non-PC groups (*P* = 0.378), with 31 and 75 deaths, respectively. Patients with PC had smaller tumours and were more likely to receive sublobar resection and to be operated on with a minimally invasive approach. Previous gastric cancer (*P* = 0.042) and synchronous PC (when diagnosed up to 6 months before lung adenocarcinoma; *P* = 0.044) were related, with a worse OS. Colon and breast adenocarcinomas and melanomas were significantly related to a lower incidence of high grade (solid or micropapillary, *P* = 0.0039, *P* = 0.005 and *P* = 0.028 respectively), whereas patients affected by a previous lymphoma had a higher incidence of a micropapillary pattern (*P* = 0.008).

**CONCLUSIONS:**

In patients with PC, we found smaller tumours more frequently treated with minimally invasive techniques and sublobar resection, probably due to a more careful follow-up. The impact on survival is not uniform and predictable; however, breast and colon cancers and melanoma showed a lower incidence of solid or micropapillary patterns whereas patients with lymphomas had a higher incidence of a micropapillary pattern.

**Figure:**
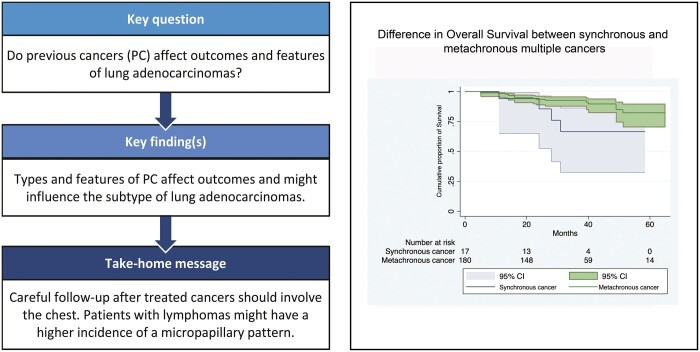

## INTRODUCTION

Lung cancer is the leading cause of death of cancer worldwide, and adenocarcinoma is the most frequent histotype [1]. In 2011, the International Association for the Study of Lung Cancer/American Thoracic Society/European Respiratory Society classification [2] redefined several different patterns of lung adenocarcinomas, which differed not only according to their pathological features, but also according to their clinical behaviours, with a crucial impact on long-term outcomes, as demonstrated in several reports [3].

Epidemiology reports showed that an increasing number of patients are diagnosed with >1 cancer beyond lung cancer in their lifetime, due to the increased life expectancy, improved cancer treatments and more careful surveillance [4–7]. A population-based study [8] reported that lung cancer was the most frequently diagnosed second primary cancer among cancer survivors in the USA. A frequency of up to 22% has been described so far [9], but the exact percentage of patients with multiple cancers varies widely because the different reports based their selection of patients and their surveillance protocol on the type of cancer and on the respective reimbursement systems of each national health system. Moreover, differences in ethnicity in the analysed cohort of patients also have a strong influence on the epidemiology of cancers [10]. Nevertheless, the evidence is not consistent enough to be used effectively to define the risk profiles of these patients; moreover, a possible correlation between previous cancers (PC) and the adenocarcinoma pattern has not been fully investigated.

The goal of the present study was to assess the features of patients diagnosed with early-stage lung adenocarcinomas with PC and to verify a possible correlation between PC and adenocarcinoma histological subtypes.

## MATERIAL AND METHODS

### Ethic statement

This study was approved by the ethical committee of Verona and Rovigo, Italy, on 13 February 2019 (protocol number 8543). Written consent was obtained from the participants if possible, according to current regulations.

### Patients

We retrospectively collected data from all consecutive patients with pathological stage I and II lung adenocarcinomas operated on between January 2014 and December 2017 in 9 European thoracic surgery departments. Seven Italian institutions (IRCCS Sacro Cuore don Calabria Hospital in Negrar di Valpolicella, Verona; University Hospital of Parma; University Hospital of Pisa; University Hospital of Varese; University of Sacred Heart, IRCCS Fondazione Policlinico Agostino Gemelli in Rome; IRCCS Arcispedale Santa Maria Nuova, Reggio Emilia; University Hospital of Turin), 1 Spanish (Clinica Universidad de Navarra) and 1 Swiss (Cantonal Hospital Lucerne) participated in this study.

All patients with complete preoperative data and follow-up information were included in this study.

All patients should have undergone pulmonary resection (either anatomical or non-anatomical) with radical intent and lymph node dissection, but the surgical technique (either open or minimally invasive) was based on each institution’s preference.

### Histological classification of lung adenocarcinoma

All cases were diagnosed according to the International Association for the Study of Lung Cancer/American Thoracic Society/European Respiratory Society classification system, and adenocarcinoma subtypes were recorded semiquantitatively in 5% increments by pathologists at each institution. Diagnoses were reached by consensus among pathologists at the same institution that were blinded to patient outcomes. All cases were staged according to the 8th edition of the International Union Against Cancer/American Joint Committee on Cancer TNM classification.

In the analysis, a pattern was considered to be present if it was the predominant or the second predominant pattern.

### Previous cancers

Patients were divided according to the presence or absence of PC. We then analysed the incidence of the different lung adenocarcinoma patterns. A PC occurring up to 6 months before a lung adenocarcinoma was defined as synchronous; if the PC occurred beyond 6 months, it was defined as metachronous. Warren and Gates criteria [11] were used to identify PC; moreover, patients with previous lung cancer should have fit the modified Martini and Melamed criteria [12–14]: different histological diagnosis or a latency of at least 2 years in cases of similar histological diagnoses, tumours located in different lobes, and no record of positive lymph nodes or evidence of distant metastases. All PC should have been radically treated.

### Statistical analyses

Data were analysed using the SPSS software version 26.0 for IOS (SPSS, Chicago, IL, USA). Continuous variables were expressed as the mean with the standard deviation or the median with a range, whereas categorical variables were expressed in terms of frequency. Logistic regression was used for inter-group comparison of categorical variables, whereas analysis of variance was used for continuous variables. Time to disease relapse (TDR) was defined as the time from the day of the operation until the first evidence of relapse whereas patients without a recurrence were censored at their last follow-up. Overall survival (OS) was considered the time from the day of the operation for the most recent lung adenocarcinoma until death from any cause; patients who did not die were censored at their last follow-up. Survival and time to relapse were estimated with the Kaplan–Meier method, and differences in survival were determined by log-rank analysis.

## RESULTS

**Figure 1:**
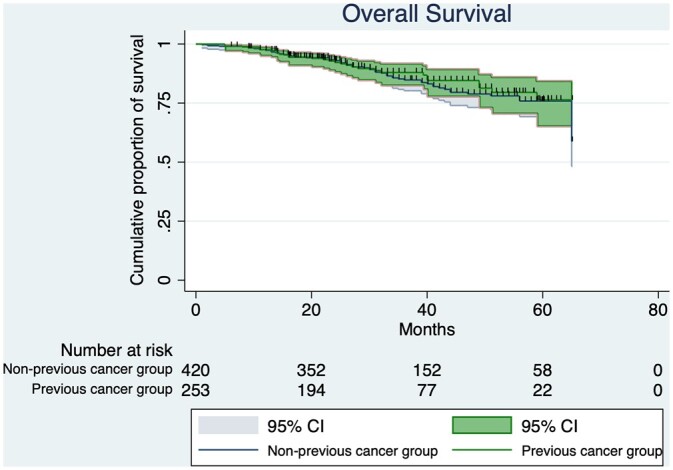
Overall survival of patients with and without previous cancers affected by lung adenocarcinoma.

We included 700 patients in the study. All preoperative, intraoperative and postoperative characteristics are listed in Table 1. A total of 260 patients (37.1%) had a PC. The median time from the diagnosis of the first cancer and the lung adenocarcinoma was 51 months (range 1–494). In 17 cases (2.4%), PC were synchronous with lung cancer. When compared with patients with no PC, patients who had a PC presented a significantly lower maximal standard uptake value of the tumour (7.7 vs 6.0; *P* = 0.006); they were more likely to undergo a wedge resection rather than an anatomical resection (*P* > 0.001); a higher proportion of them had minimally invasive surgery (*P* = 0.029); and they had significantly smaller tumours (25.0 vs 21.9 mm; *P* = 0.002). The median follow-up time for the entire cohort was 52 months (range 24–68). The 2 groups did not differ either in terms of OS or TDR. We observed 31 deaths in the PC group and 75 deaths in the non-PC group; the 1-, 3- and 5-year OS for the group with and without PC was 97.1%, 84.6% and 75.7% and 97.6%, 88.0% and 76.4%, respectively (*P* = 0.378; Fig. 1). In the PC group, we observed 63 recurrences, whereas there were 113 recurrences in the non-PC group; the 1-, 3- and 5-year TDR for the groups with and without PC was 90.4%, 72.4% and 64.8% and 94.1%, 74.1% and 62.4, respectively (*P* = 0.938). All patients had an R0 resection.

**Table 1:** Preoperative, intraoperative and postoperative features of patients in the cohort and a comparison of subgroups with and without previous cancers

	Entire cohort	No previous cancers (*n* = 440)	Previous cancers (*n* = 260)	*P*-Value
Gender, *n* (%)				
Male	391 (55.9)	246 (55.9)	145 (55.8)	0.971
Age, mean (range)	68.4 (41–91)	68.1 (41–90)	68.8 (42–91)	0.282
Cardiovascular comorbidities, *n* (%)				
Yes	423 (60.4)	266 (60.5)	157 (60.4)	0.995
Smoking history, *n* (%)				
Yes	511 (73)	316 (71.8)	195 (75.0)	0.181
Diabetes, *n* (%)				
Yes	97 (13.8)	55 (12.5)	42 (16.2)	0.179
Respiratory comorbidities, *n* (%)				
Yes	191 (27.3)	118 (26.8)	73 (28.1)	0.709
ASA score, *n* (%)				0.090
1	100 (14.3)	68 (15.5)	32 (12.3)	
2	362 (51.7)	230 (52.3)	132 (50.9)	
3	187 (26.7)	110 (25.0)	77 (29.6)	
4	18 (2.5)	7 (1.6)	11 (4.2)	
SUVmax T, mean (±SD)	7.0 (±5.9)	7.7 (±6.5)	6.0 (±4.7)	**0.006**
FEV1%, mean (±SD)	95.6 (±21.6)	95.5 (±22.0)	95.8 (±21.1)	0.859
DLCO%, mean (±SD)	74.3 (±28.4)	73.1 (±28.7)	76.1 (±28.0)	0.351
Side of the lung adenocarcinoma, *n* (%)				
Right	409 (58.4)	258 (58.6)	151 (58.1)	0.885
Site of the lung adenocarcinoma, *n* (%)				
Upper lobe	411 (58.7)	264 (60.0)	147 (56.5)	0.504
Middle lobe	46 (6.6)	29 (6.6)	17 (6.5)	
Lower lobe	214 (30.6)	126 (28.6)	88 (33.9)	
More than 1 lobe	29 (4.1)	21 (4.8)	8 (3.1)	
Lung resection, *n* (%)				**>0.001**
Wedge resection	60 (8.6)	25 (5.7)	35 (13.5)	
Anatomical segmentectomy	72 (810.3)	40 (9.1)	32 (12.3)	
Lobectomy	556 (79.5)	364 (82.7)	192 (73.8)	
Bilobectomy/pneumonectomy	12 (1.8)	11 (2.5)	1 (0.4)	
Surgical technique, *n* (%)				**0.029**
Open	406 (58.0)	269 (61.1)	137 (52.7)	
VATS	252 (36.0)	144 (32.7)	108 (41.5)	
Robotic	42 (6.0)	27 (6.1)	15 (5.8)	
Presence of pattern in lung adenocarcinoma, *n*				
Lepidic	292	175	117	0.176
Acinar	495	311	184	0.980
Papillary	247	158	89	0.653
Solid	195	131	64	0.142
Micropapillary	88	50	38	0.211
Mucinous	38	27	11	0.285
Lymphovascular invasion, *n* (%)				
Yes	131 (18.7)	80 (18.2)	51 (19.6)	0.881
Pleural invasion, *n* (%)				
Yes	217 (1.0)	142 (32.3)	75 (28.9)	0.343
Size of the tumour, mm mean (±SD)	23.9 (±12.7)	25.0 (±13.1)	21.9 (11.9)	**0.002**
N status, *n* (%)				0.865
N0	625 (89.3)	392 (89.1)	225 (86.5)	
N1	75 (10.7)	48 (10.9)	27 (10.4)	
pStage, *n* (%)				0.197
Stage I (A1, 2, 3 and B)	532 (76.0)	327 (74.3)	205 (78.9)	
Stage II (A and B)	161 (23.0)	108 (24.6)	53 (20.4)	

ASA: American Society of Anesthesiology; DLCO%: diffusing capacity of the lungs for carbon monoxide; FEV1%: forced expiratory volume in 1 s; SD: standard deviation; SUVmax: maximum standard uptake value; VATS: video-assisted thoracoscopic surgery. Bold values are statistically significant.

To verify the prognostic impact of PC on OS and TDR, we performed univariable and multivariable analyses, including the most important prognostic factors (Table 2). The presence of PC did not have a significant prognostic impact on OS or on TDR.

**Table 2:** Univariable and multivariable analyses for possible prognostic factors

Variable	Overall survival				Disease-free interval			
	Univariable		Multivariable		Univariable		Multivariable	
	*P*-Value	HR (95% CI)	*P*-Value	HR (95% CI)	*P*-Value	HR (95% CI)	*P*-Value	HR (95% CI)
Age	**0.002**	**1.040 (1.015–1.065)**	**0.043**	**1.043 (1.001–1.085)**	**0.021**	**1.021 (1.003–1.040)**	0.219	1.016 (0.991–1.042)
Male gender (versus female)	**0.001**	**1.972 (1.302**–**2.987)**	1.586	1.012 (0.845–2.979)	**0.039**	**1.377 (1.016–1.866)**	0.131	1.381 (0.908–2.098)
Smoker	0.071	1.617 (0.960–2.726)			0.096	1.382 (0.944–2.022)		
Previous cancer	0.382	0.829 (0.544–1.263)	0.977	0.990 (0.521–1.883)	0.938	0.988 (0.726–1.345)	0.917	1.023 (0.666–1.572)
SUVmax	**<0.001**	**1.067 (1.032–1.104)**	**0.002**	**1.061 (1.022–1.100)**	**<0.001**	**1.059 (1.034–1.085)**	**<0.001**	**1.050 (1.022–1.079)**
Anatomical resection (versus wedge)	0.419	1.405 (0.616–3.207)			0.627	0.880 (0.526–1.472)		
Minimally invasive (versus open)	**0.044**	**0.633 (0.406–0.987)**	0.901	1.042 (0.545–1.990)	0.067	0.746 (0.545–1.021)		
Lymphovascular invasion	0.508	1.193 (0.707–2.015)			0.327	1.213 (0.825–1.782)		
Pleural invasion	0.164	1.322 (0.892–1.957)			0.156	1.252 (0.918–1.707)		
Size of the tumour	**0.003**	**1.020 (1.007–1.034)**	0.461	1.008 (0.986–1.031)	**<0.001**	**1.024 (1.013–1.034)**	**0.001**	**1.023 (1.009–1.038)**

CI: confidence interval; HR: hazard ratio; SUVmax: maximum standard uptake value. Bold values are statistically significant.

Table 3 reports the most frequent site of PC. Breast adenocarcinoma was the most frequent type of cancer (56 cases) followed by lung cancer, prostate adenocarcinoma and colon adenocarcinoma (30, 29 and 28 cases, respectively). Fourteen patients had >1 type of cancer prior to the lung adenocarcinoma.

**Table 3:** Site, histological diagnosis, incidence and number of events of patients with previous cancers

Site of previous cancer	Number of patients	Percentage of the whole cohort	Percentage among patients with previous cancers	Number of deaths	Number of recurrences
Breast adenocarcinoma	56	8.0	21.5	5	11
Lung	30	4.3	11.5	2	9
Prostate adenocarcinoma	29	4.1	11.2	5	11
Colon adenocarcinoma	28	4.0	10.8	5	7
Other	21	3.0	8.1	3	3
Larynx squamous cell carcinoma	15	2.1	5.8	3	4
Lymphoma	14	2.0	5.4	3	5
More than 1	14	2.0	5.4	1	4
Bladder	13	1.9	5.0	4	4
Thyroid	12	1.7	4.6	0	2
Kidney	8	1.1	3.1	0	3
Melanoma	6	0.9	2.3	0	1
Gastric adenocarcinoma	5	0.7	1.9	1	0
Uterus	5	0.7	1.9	0	1
Hepatocarcinoma	4	0.6	1.5	0	1
Total	260	37.1	100.0	31	63

Most patients (511, 73%) had a history of smoking (either a former or a current smoker at the time of the lung adenocarcinoma diagnosis). Patients with colon cancer were more likely to be smokers compared to all the other patients (*P* = 0.025), whereas those who previously had a breast cancer had a lower incidence of smoking history (*P* = 0.001).

**Figure 2:**
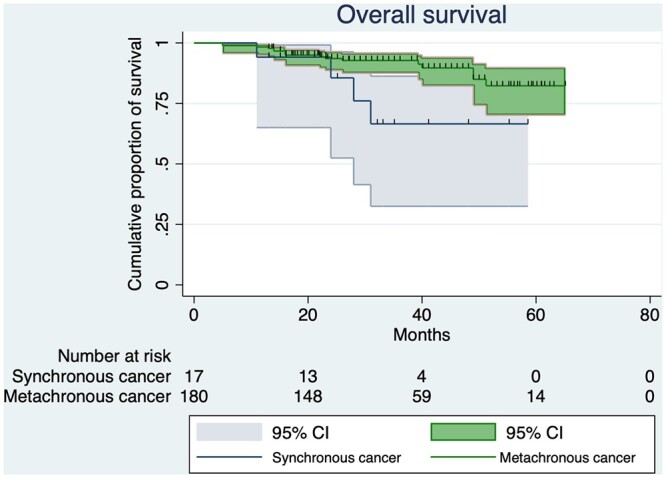
Overall survival of patients with synchronous or metachronous multiple cancers.

The 17 patients with synchronous lung adenocarcinoma and PC had a significantly worse OS compared to those with metachronous lung adenocarcinoma (47.2 vs 59.5; *P* = 0.044; Fig. 2). We analysed possible extensions of the cut-off for synchronous cancer: When we used a 9-month cut-off (29 patients with synchronous cancers), OS was still significantly different between the 2 groups (*P* = 0.027), whereas statistical significance was lost when the cut-off was set as 12 months (38 synchronous cancers; *P* = 0.121).

We did not find any significant difference in OS or TDR according to the type of cancer, except in patients who had a previous gastric adenocarcinoma who had a worse OS both when compared to the rest of the whole cohort (57.2 vs 39.5 months; *P* = 0.042) and to the group of patients with PC (58.1 vs 39.5 months; *P* = 0.023).

### Previous lung cancer

We performed a subanalysis of 34 patients with a previous lung cancer (30 with only a previous lung cancer and 4 with more than 1 previous malignancy). In 7 cases, information regarding histological analysis, date of diagnosis and therapy of previous lung cancer was not available. The histological diagnoses of the PC were as follows: 18 adenocarcinomas, 1 adenosquamous carcinoma, 1 small-cell lung cancer (SCLC), 4 squamous cell carcinomas, 2 typical carcinoids and 1 atypical carcinoid. Surgery was the treatment of choice for all patients except 2, who were treated with chemotherapy (1 of whom was affected by SCLC) and chemoradiation, respectively. One patient received neoadjuvant therapy, 2 patients underwent adjuvant treatment and 1 had both neoadjuvant and adjuvant. Among 21 patients with information regarding the site of the first tumour, 13 were on the same side as the subsequent lung adenocarcinoma. The mean time from the first diagnosis to the second was 55.13 months (range 10–154 months). The radiological features of the ‘second’ lung adenocarcinomas were primarily solid or part solid nodules (29, 85.3%), whereas only 5 patients were reported to have a ground-glass nodule. In this subpopulation, we could appreciate a significantly higher proportion of sublobar resections compared to the other patients with non-pulmonary PC (36.4% vs 24.7%; *P* < 0.001).

### Correlation between lung adenocarcinoma subtypes and previous cancers

We analysed a possible correlation between the type of PC and the patterns of the lung adenocarcinomas. Our analysis showed that patients with a previous colon adenocarcinoma and a breast adenocarcinoma had a significantly lower incidence of solid lung adenocarcinomas (*P* = 0.039 and *P* = 0.005, respectively). Concurrently, patients with previous melanomas had a significantly lower incidence of the micropapillary predominant pattern (*P* = 0.028) and a higher incidence of the papillary pattern (*P* = 0.013); the papillary pattern was also more frequent in patients affected by a previous bladder malignancy. Moreover, previous thyroid cancer was related to a lower possibility of developing a mucinous pattern lung adenocarcinoma (*P* = 0.003), whereas stomach adenocarcinoma showed a higher percentage of lepidic pattern lung adenocarcinoma (*P* = 0.038). Lastly, patients who had a previous lymphoma had a significantly higher risk of developing a micropapillary-pattern lung adenocarcinoma (*P* = 0.008).

## DISCUSSION

Cancer is one of the leading causes of death worldwide. Among all type of cancers, lung cancer is one of the most lethal [1]. Moreover, in recent decades, lung adenocarcinoma has become the most frequent histotype, overcoming squamous cell carcinoma.

It is not rare that patients diagnosed with lung cancer report previous malignancies, even though the real incidence of PC in these patients is not homogeneously and consistently reported in the literature and might vary up to 26% [15, 16]. The large variation in this data is probably due to the selection criteria and ethnic features of the patient’s cohort. In our study, 260/700 (37.1%) had a history of other cancers before lung adenocarcinoma. We can speculate that these data, which seem higher than those reported previously, are due to the highly selected nature of our cohort (early-stage, surgically resected lung cancer). Moreover, breast adenocarcinoma, lung cancer and prostate adenocarcinoma accounted for the most represented PC; these data are in line with those reported by Song and colleagues [16] based on the Surveillance, Epidemiology and End Results database. Conversely, Liu *et al.* [17] and Donin *et al.* [18] found a higher proportion of cancers from the upper aerodigestive tract, whereas Ventura *et al.* [9] reported a higher incidence of cancers from the urogenital system.

Patients with PC showed peculiar characteristics related to the anatomical features of lung adenocarcinoma and its treatment. In fact, these patients had significantly smaller tumours, and they were more likely to have a minimally invasive surgical approach; on the other hand, sublobar, non-anatomical resections were significantly higher in this group compared to patients who had no previous malignancies. Minimally invasive surgery has been demonstrated to reduce postoperative complications and pain and improve quality of life [19]. It is now considered the standard of care for the treatment of early-stage lung cancer; nevertheless, the use of video-assisted thoracoscopic surgery or robot-assisted thoracic surgery is strictly related to the surgeon’s skills, experience and case-based preferences. Due to the faster postoperative course, it is possible that the minimally invasive approach has been preferred in frailer patients, such as those who have already undergone previous operations, radiotherapy or systemic treatments. Similarly, in these patients, we noticed a higher tendency towards wedge resection. To date, lobectomy is still considered the standard of care for primary lung cancer [20, 21], even though sublobar anatomical resections are gaining a wider consent for SCLC [22, 23]. Nevertheless, segmentectomies require some additional technical skills, especially if performed with minimally invasive techniques. Although the proportion of wedge resections was significantly higher in the group of patients with previous lung cancer, we appreciate that this difference was particularly significant in patients with a previous lung cancer. In fact, the main goal of a sublobar resection is to spare lung tissue to preserve pulmonary function. In patients who underwent a previous lung resection, wedge resection was used as an alternative to lobectomy to avoid severe detriment to lung function.

It has been well established that synchronous multiple cancers have worse outcomes compared to metachronous tumours [9, 24, 25]. In our cohort, despite the fact that a majority of the patients had a metachronous tumour, the results were consistent with these observations. In a meta-analysis regarding patients with multiple lung cancers, Jiang *et al.* [26] reported that no significant difference in OS could be observed when survival was measured from the treatment of the second cancer. Conversely, we found a significant difference in survival even if we counted the period from the surgical resection of the lung cancer (the second cancer). In the aforementioned studies, cancers are considered synchronous when the diagnosis of the second cancer is made within 6 months from the diagnosis of the first one. We explored the potential extensions of this cut-off, and we found that when we considered a 9-month cut-off, the difference in OS between the 2 groups was even more significant, whereas no difference in survival was seen if a 1-year cut-off was considered. Based on these results, we observed that the detrimental effect of synchronous cancers is still significant beyond the 6-month cut-off; these patients probably require more careful surveillance and dedicated treatments. We might speculate that this difference in survival is related to the earlier diagnosis of a metachronous new primary cancer, which might allow more radical therapies.

Conversely, we did not observe differences in long-term outcomes between patients with or without PC. In the subgroup analysis, only patients with a previous gastric adenocarcinoma had significantly worse OS, even if the small size of this subgroup might have influenced the results. Conversely, Huang *et al.* [27] analysed the outcomes of patients with lung cancer after a previous hepatogastrointestinal cancer and found an increased OS compared to other types of cancers, but no subanalysis was conducted to verify outcomes of patients who had only a gastric cancer.

The evidence in the literature confirmed that high-grade patterns of lung adenocarcinoma, namely micropapillary and solid, have worse outcomes, both in terms of OS and TDR, compared to other subtypes [3]. To the best of our knowledge, our study is the first to correlate the incidence of adenocarcinoma subtype and the histological diagnosis of PC. We hypothesized that the prevalence of each lung adenocarcinoma histotype could be related to genetic factors that might also influence the development of other cancers. In our analysis, colon and breast adenocarcinoma and melanoma seem to have a ‘protective’ effect because we found a lower incidence of solid and micropapillary subtypes. On the other hand, patients with a previous lymphoma have a higher tendency to develop a micropapillary lung adenocarcinoma. The clinical consequence of these results might be the standardization of different surveillance paths with a more careful test of lung parenchyma. A worse outcome for patients treated for non-small-cell lung cancer and affected by a previous lymphoma has already been reported [28, 29]. In our cohort, despite the higher prevalence of the micropapillary pattern in this subgroup, we did not find significantly worse survival, but these results might be explained by the low number of patients. In addition, a large Italian study [30] analysed the outcomes of patients treated for non-small-cell lung cancer with previous lymphoma, finding a higher incidence of adenocarcinomas compared to other histological types; the authors confirmed the feasibility and safety of surgery in these patients.

As already mentioned, our population had a more careful follow-up because they had PC. Nevertheless, patient follow-up after radical treatment of malignancy may vary according to the type of cancer, the pathological stage and the national or international follow-up guidelines. Chest imaging is not always recommended. Moreover, adherence to guidelines might be limited by institutional protocols or personal beliefs [31, 32]. Recent studies stressed that chest CT imaging does not add any benefit in patients affected by early-stage breast cancer [33] and after hepatocellular carcinoma [34], whereas it might be worthwhile in selected patients who have melanoma [35]; on the other hand, a chest CT scan is suggested for patients operated on for lung cancer. This suggestion was recently confirmed by Mitchell and colleagues [36].

We believe that, although one cannot draw final conclusions from the findings of our study, the results suggest a more careful chest follow-up in a selected group of patients with a higher risk to develop more aggressive adenocarcinoma patterns. These patients could potentially also be considered for dedicated lung cancer screening.

### Limitations

Our study has some limitations. First, it is limited by its retrospective nature; second, lung adenocarcinoma specimens were reviewed by each institution, without a concordance analysis. Moreover, the molecular biological characteristics of the tumours were not homogeneously analysed among the different institutions, so that information could not be used in the analysis of this paper. Lastly, missing data regarding primary tumour details might have influenced the final results of the paper.

## CONCLUSIONS

In a broad cohort of patients with early-stage, radically resected lung adenocarcinomas, the presence of PC did not have a strong influence on OS, and only patients with a previous gastric adenocarcinoma showed worse survival. Concurrently, patients with synchronous cancers had the worst outcomes, but the cut-off should be extended to up to 9 months.

Patients with multiple cancers showed peculiar features related to lung adenocarcinoma and its treatment: They had smaller tumours and had more sublobar resections. Lastly, our research highlighted a significant correlation between some types of tumours and lung adenocarcinoma patterns that might lead to different follow-up protocols if our data can be validated in larger prospective studies.

## Funding

This research did not receive any specific grants from funding agencies in the public, commercial or not-for-profit sectors.

**Conflict of interest:** none declared.

## Data Availability Statement

The data underlying this article cannot be shared publicly due to privacy and ethical reasons.

## Author contributions

**Pietro Bertoglio:** Conceptualization; Data curation; Formal analysis; Investigation; Methodology; Project administration; Supervision; Validation; Visualization; Writing—original draft. **Luigi Ventura:** Data curation; Investigation; Validation; Writing—review & editing. **Vittorio Aprile:** Data curation; Formal analysis; Investigation; Supervision; Validation; Writing—review & editing. **Maria Angela Cattoni:** Data curation; Investigation; Validation; Writing—review & editing. **Dania Nachira:** Data curation; Investigation; Validation; Visualization; Writing—review& editing. **Filippo Lococo:** Data curation; Investigation; Supervision; Validation; Visualization; Writing—review & editing. **Maria Rodriguez Perez:** Data curation; Investigation; Supervision; Validation; Visualization; Writing—review & editing. **Francesco Guerrera:** Data curation; Investigation; Supervision; Validation; Visualization; Writing—review & editing. **Fabrizio Minervini:** Data curation; Investigation; Validation; Visualization; Writing—review & editing. **Letizia Gnetti:** Data curation; Investigation; Visualization; Writing—review & editing. **Alessandra Lenzini:** Data curation; Investigation; Visualization; Writing—review & editing. **Francesca Franzi:** Data curation; Investigation; Supervision; Validation; Visualization; Writing—review & editing. **Giulia Querzoli:** Conceptualization; Data curation; Investigation; Validation; Visualization; Writing—review & editing. **Guido Rindi:** Data curation; Investigation; Supervision; Validation; Visualization; Writing—review & editing. **Salvatore Bellafiore:** Data curation; Investigation; Resources; Validation; Visualization; Writing—review & editing. **Federico Femia:** Data curation; Investigation; Validation; Visualization; Writing—review& editing. **Giuseppe Salvatore Bogina:** Data curation; Investigation; Supervision; Validation; Visualization; Writing—review & editing. **Diana Bacchin:** Data curation; Investigation; Validation; Writing—review & editing. **Peter Kestenholz:** Data curation; Investigation; Supervision; Validation; Writing—review & editing. **Enrico Ruffini:** Data curation; Methodology; Supervision; Validation; Visualization; Writing—review & editing. **Massimiliano Paci:** Data curation; Investigation; Supervision; Validation; Visualization; Writing—review & editing. **Stefano Margaritora:** Data curation; Investigation; Methodology; Supervision; Validation; Visualization; Writing—review & editing. **Andrea Selenito Imperatori:** Data curation; Investigation; Methodology; Supervision; Validation; Visualization; Writing—review & editing. **Marco Lucchi:** Data curation; Investigation; Methodology; Supervision; Validation; Visualization; Writing—review & editing. **Luca Ampollini:** Data curation; Investigation; Supervision; Validation; Visualization; Writing—review & editing. **Alberto Claudio Terzi:** Conceptualization; Data curation; Investigation; Methodology; Supervision; Validation; Visualization; Writing—original draft.

## Reviewer information

Interactive CardioVascular and Thoracic Surgery thanks Alessandro Gonfiotti, David G. Healy and the other, anonymous reviewer(s) for their contribution to the peer review process of this article.

## Notes

Presented at the 57th Virtual Congress of the Society of Thoracic Surgeons, 29–31 January 2021.

## ABBREVIATIONS

OS: Overall survival
PC: Previous cancer
SCLC: Small-cell lung cancer
TDR: Time to disease relapse
